# SnO_2_ Nanostructures: Effect of Processing Parameters on Their Structural and Functional Properties

**DOI:** 10.1186/s11671-017-2100-2

**Published:** 2017-05-04

**Authors:** Tetiana A. Dontsova, Svitlana V. Nagirnyak, Vladyslav V. Zhorov, Yuriy V. Yasiievych

**Affiliations:** Department of Chemistry, National Technical University of Ukraine “Igor Sikorsky KPI”, Kyiv, 03056 Ukraine

**Keywords:** Tin (IV) oxide, Thermal evaporation method, 0D nanostructures, 1D nanostructures, I–V curves

## Abstract

Zero- and 1D (one-dimensional) tin (IV) oxide nanostructures have been synthesized by thermal evaporation method, and a comparison of their morphology, crystal structure, sorption properties, specific surface area, as well as electrical characteristics has been performed. Synthesized SnO_2_ nanomaterials were studied by X-ray diffraction, scanning and transmission electron microscopy (SEM and TEM), N_2_ sorption/desorption technique, IR spectroscopy and, in addition, their current-voltage characteristics have also been measured. The single crystalline structures were obtained both in case of 0D (zero-dimensional) SnO_2_ powders and in case of 0D nanofibers, as confirmed by electron diffraction of TEM. It was found that SnO_2_ synthesis parameters significantly affect materials’ properties by contributing to the difference in morphology, texture formation, changes in IR spectra of 1D structure as compared to 0D powders, increases in the specific surface area of nanofibers, and the alteration of current-voltage characteristics 0D and 1D SnO_2_ nanostructures. It was established that gas sensors utilizing of 1D nanofibers significantly outperform those based on 0D powders by providing higher specific surface area and ohmic I–V characteristics.

## Background

Tin (IV) oxide (SnO_2_) is a typical *n*-type semiconductor with a wide direct band gap of 3.6 eV [1]. SnO_2_ exhibits a number of interesting functional properties such as optical transparency in the visible spectrum [2], chemical stability at high temperatures [3], good surface adsorption properties of oxygen and availability of numerous oxygen species and active acid sites on its surface [4], high specific theoretical capacity [5], and excellent electrical characteristics [3, 6]. As a result, SnO_2_ is broadly used as a part of catalysts for oxidation of organic compounds [4, 7], as an anode material in lithium-ion batteries [5], as transparent electrodes in solar cells [8], as a host material and a buffer layer in many optoelectronic devices [9], or as a sensitive layer in gas sensors to detect harmful for human health and hazardous gases such as CO, NO_*x*_, H_2_S, H_2_, and CH_4_. [10–13]. Today, the development of superior gas sensors is extremely important because they not only allow safely controlling the environment at home and industrial settings [12] but also provide an easy diagnostic tool for detection of early stages of otherwise hard or impossible to detect diseases at air exhalation among other applications [14].

It was established [15] that nanostructured SnO_2_ provides far better gas sensing properties as compared to SnO_2_ micron size materials. Thermal evaporation [16], hydrothermal synthesis [17], sol-gel method [18, 19], template synthesis [20], and laser ablation [21] are the most explored methods for synthesis of SnO_2_ nanostructures. Thermal evaporation method is the most promising technique as it allows to produce single crystalline 0D (zero-dimensional) or 1D (one-dimensional) SnO_2_ nanoparticles with high specific surface area and excellent gas sensing properties [16, 22].

There are many papers recently published that study either 0D or 1D nanostructured SnO_2_ [15, 16, 23, 24]. However, the direct comparison of performance of these structurally very different materials is lacking. Therefore, the goal of this paper is to fill the gap by providing a comparison of structural and functional behavior of 0D and 1D SnO_2_ nanostructures.

## Methods

### Materials Synthesis

Tin (II) oxalate (SnC_2_O_4_), prepared by sol-gel method from tin (II) chloride (SnCl_2_) and ammonium oxalate ((NH_4_)_2_C_2_O_4_) as in [10], was used as a precursor for the SnO_2_ synthesis. For the synthesis of 0D and 1D SnO_2_ nanostructured materials, 2 g of SnC_2_O_4_ powder was loaded into 50 × 10 × 10 mm alumina boat each, which were placed inside of a quartz tube in two different horizontal-type furnaces. Two furnaces had identical settings with the only exception that one furnace provided faster heating rate of 80 K/min, and a second furnace provided only 20 K/min heating rate. The N_2_ gas with 0.005% O_2_ impurity content was used as an inert atmosphere that was supplied to the quartz tube before heating began [10, 22]. Both furnaces were heated to 1123 K and kept at this temperature for dwell time of 1 h. After finishing of synthesis procedure, furnaces were turned off and cooled naturally. As a result of the thermal evaporation, the nanocrystalline SnO_2_ was obtained due to following reactions [22]:

$$ \begin{array}{l}\mathrm{Sn}{\mathrm{C}}_2{\mathrm{O}}_4\to \mathrm{Sn}\mathrm{O} + \mathrm{C}\mathrm{O} + \mathrm{C}{\mathrm{O}}_2,\\ {}\mathrm{Sn}\mathrm{O}\ \to\ \mathrm{Sn}{\mathrm{O}}_2 + \mathrm{Sn},\\ {}\mathrm{Sn} + {\mathrm{O}}_2\to\ \mathrm{Sn}{\mathrm{O}}_2.\end{array} $$

The SnO_2_ sample with the fast heating rate was marked as TO1, and the SnO_2_ sample with slow heating rate was named TO2.

### Characterization Techniques

In X-ray diffractometer Ultima IV (Rigaku, Japan) with CuКα radiation at 40 kV, 30 mA was used to collect diffraction patterns of the SnO_2_ samples. The powdered samples were scanned from 20 to 80 2θ at 1°/min with a scanning step of 0.0001°. XRD patterns were analyzed by the PDXL software package using database ICDD/PDF-2 and COD. The crystalline size and lattice parameters of the materials were calculated automatically by the software.

Both Transmission Electron Microscopy PEM 100–01 (Selmi, Ukraine) and Scanning Electron Microscopy REM 106I (Selmi, Ukraine) were used for characterization of particle’s size and morphology of the obtained SnO_2_ samples.

Specific surface area of the samples was studied by adsorption/desorption of nitrogen (Quantachrome® Autosorb, Quantachrome Instruments, USA) using Langmuir isotherm and Brunauer-Emmett-Teller (BET)-based software.

IR 4000–400 cm^−1^ wavenumber spectra of SnO_2_ were collected using FTIR spectrometer (Thermo Nicolet Nexus FTIR, Thermo Fisher Scientific, USA). For spectra collection, SnO_2_ samples were mixed with pre-dried KBr (for spectroscopy, “Aldrich,” USA) at 1:30 SnO_2_/KBr ratio.

**Fig. 1:**
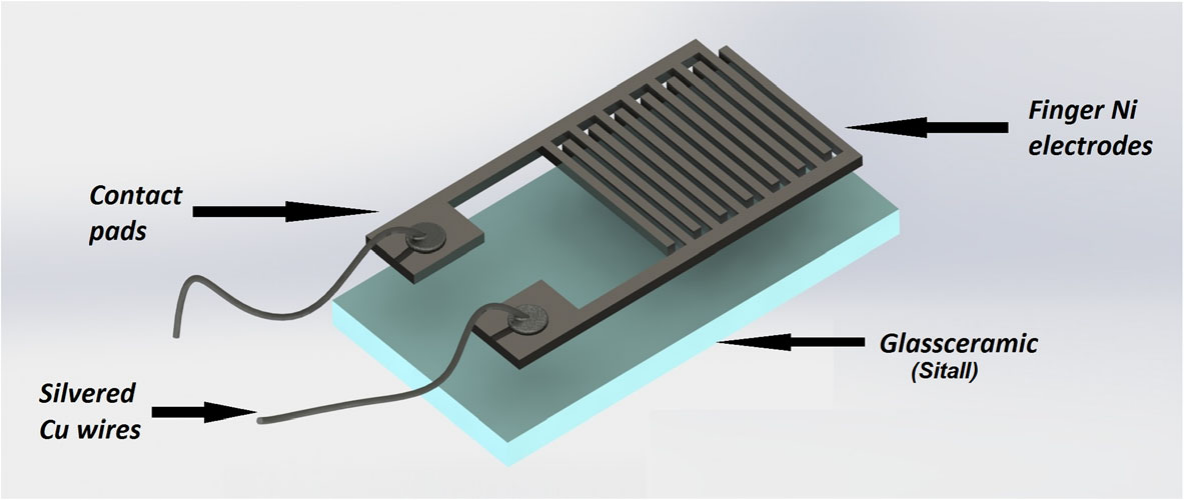
A schematic presentation of gas sensor’s test structure used for I–V measurements of SnO_2_

Measurements of current-voltage characteristics of SnO_2_ nanostructures were carried by using gas sensors developed utilizing a special test structures. A 6 × 10 × 2 mm crystalline glass ceramic material (Sitall, Ukraine) [25] was used as a substrate upon which the interdigitated Ni electrodes were deposited by a thermal evaporation technique (Fig. 1). As a result of the deposition, Ni electrodes with 50 μm width 50 and 400 μm thickness were produced. After electrode deposition, the copper wires covered with silver and coated by Teflon were connected to the contact pads by soldering using common lead-bearing solder (60% tin; 40% lead). After that, the SnO_2_ material was deposited on the top of the Ni electrodes. The deposition of sensitive layer was performed by the sedimentation on the surface of electrodes. For this purpose, SnO_2_ 1 g/cm^3^ suspension in ethanol was prepared and stirred in ultrasound bath for 90 s, after which the complete test structure with deposited interdigitated Ni electrodes, contact pads and parts of Cu wires was fully immersed into the suspension and left overnight to achieve a complete liquid evaporation. After the deposition of SnO_2_ layer on Ni-interdigitated electrodes of the test structure, the gas sensor was produced.

**Fig. 2:**
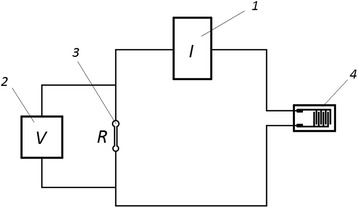
Block diagram for studying of I–V characteristics: 1—power supply; 2—voltmeter; 3—model resistance; 4—test structure of gas sensor

The block diagram of the electrical circuit with resistance connected in parallel used to measure the electrical properties of gas sensors are shown in Fig. 2. The electrical circuit consists of the power supply, voltmeter, model resistance, and actually gas sensor test structure. During current-voltage measurements, the gas sensor was placed inside of the tube furnace, and measurements were performed at three different temperatures (323, 372, and 423 K) in 5–30 V voltage range at ambient.

## Results and Discussion

### X-ray Diffraction

**Fig. 3:**
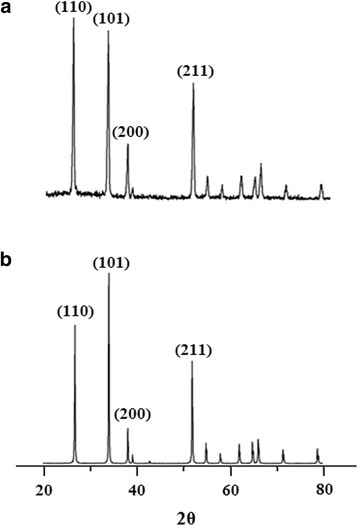
The XRD patterns of SnO_2_. **a** SnO_2_ synthesized at faster heating rate of 80 K/min (TO1). **b** SnO_2_ synthesized at slower heating rate of 20 K/min (TO2)

X-ray diffraction patterns for two SnO_2_ nanostructured materials are shown in Fig. 3, where the first diffraction pattern was collected using SnO_2_ synthesized at 80 K/min faster heating rate (Fig. 3a), while a second diffraction pattern was obtained from SnO_2_ synthesized at the same synthesis conditions, but using 20 K/min slower heating rate (Fig. 3b). As it was expected, in both cases, SnO_2_ nanostructures were crystallized in tetragonal *P42/mnm* space group with identical a = 4.74 Ǻ, c = 3,19 Ǻ lattice parameters, which correspond well with the lattice of tetragonal SnO_2_ reported in JCPDF No 41-1445 [26]. The average crystallite size calculated automatically by PDXL software from the FWHM of all peaks located between 20 and 120 2θ using Scherrer equation was equal to 80.7 and 74.3 nm for SnO_2_ with faster and slower heating rates, respectively. At the same time, the difference in heating rates during thermal evaporation synthesis affected not only crystallite size but also the X-ray peals intensity in SnO_2_ XRD patterns, especially in 1D nanostructured SnO_2_ causing texture formation resulting in the preferred orientation of the certain crystallographic directions. While isotropic material shows 100/75 ratio in (110) and (101) peaks of SnO_2_ [26], the SnO_2_ synthesized at faster heating rate of 80 K/min shows 100/95 ratio of two major *hkl* peaks; however, for SnO_2_ synthesized at slower heating rate of 20 K/min, the (110)/(101) peak intensity significantly reversed to 100/125, thus this nanostructure has a strong preferred orientation of growth in <110> crystallographic direction. Therefore, even based on the analysis of X-ray diffraction patterns of SnO_2_ synthesized at different heating rates, one can conclude that the preferred orientation of the material is different and this will certainly affect the morphology of the SnO_2_.

### Electron Microscopy

**Fig. 4:**
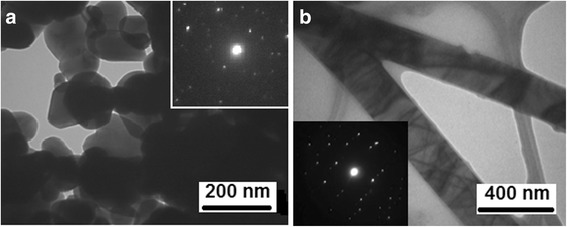
TEM images of SnO_2_ samples. **a** SnO_2_ synthesized at faster heating rate of 80 K/min (TO1). **b** SnO_2_ synthesized at slower heating rate of 20 K/min (TO2)

TEM microphotographs of SnO_2_ samples processed at two different heating rates are shown in Fig. 4. As one can see from Fig. 4, the SnO_2_ sample synthesized at 80 K/min fast heating rate (Fig. 4a) has uneven round shape of the particles with an average diameter of 50–150 nm. This measured particle size coincides perfectly with the crystallite size of 80.7 nm calculated from FWHM of the XRD peaks for this material. The selected area electron diffraction (SAED) image of the particles (Fig. 4a, insert) indicated that those are single crystalline particles. At the same time, the SnO_2_ sample synthesized at 20 K/min slow heating rate (Fig. 4b) has long and extended shapes, essentially forming 1D structures. The SAED of 1D structures also shows the crystalline nature of the material (Fig. 4b, insert), and the quality of the SAED pattern is better for 1D SnO_2_ fibers in comparison with SnO_2_ particles partially because of better crystallinity of the material and partially because only two fibers were used for SAED pattern collection, unlike for the case when many particles contributed to SAED pattern for the SnO_2_ powder.

### The Specific Surface Area

Table 1 contains results of the structural characteristics of SnO_2_ synthesized at faster heating rate of 80 K/min (TO1) and SnO_2_ synthesized at slower heating rate of 20 K/min (TO2). Pursuant to these data, the 1D SnO_2_ has specific surface area five times higher than 0D SnO_2_. Thus, the ratio of surface to volume really increases in the 1D nanostructures as compared with 0D particles.

**Table 1:** Structural characteristics of sample SnO_2_

Characteristics	TO1	TO2
S (m^2^/g)	10.6	54.8
The total pore volume (cm^3^/g)	0.076	0.065
The average conditional pore radius (nm)	144	24

**Fig. 5:**
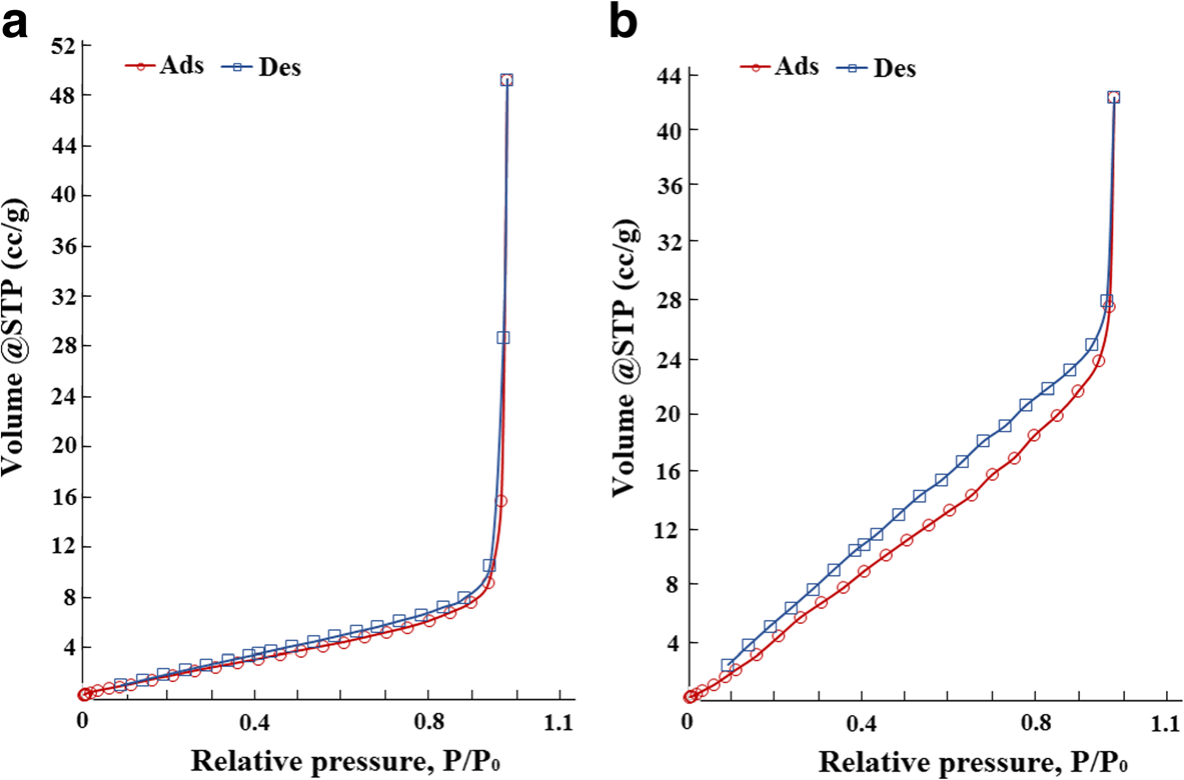
The sorption isotherm of SnO_2_ samples. **a** SnO_2_ synthesized at faster heating rate of 80 K/min (TO1). **b** SnO_2_ synthesized at slower heating rate of 20 K/min (TO2)

Also, based on the data in Table 1 and isotherms of nitrogen sorption/desorption (Fig. 5), both SnO_2_ samples are non-porous; they are consistent with the results of electron diffraction and microscopy. The revealed negligible porosity was caused by gaps between the primary particles (Table 1). In addition, the formed porous system from particles of 1D nanostructures is characterized by smaller dimensions than the porous system from 0D particles. This is evidenced by the hysteresis loop in isotherm of 1D nanostructures and the value of the average conditional pore radius for both samples.

### IR Spectroscopy

**Fig. 6:**
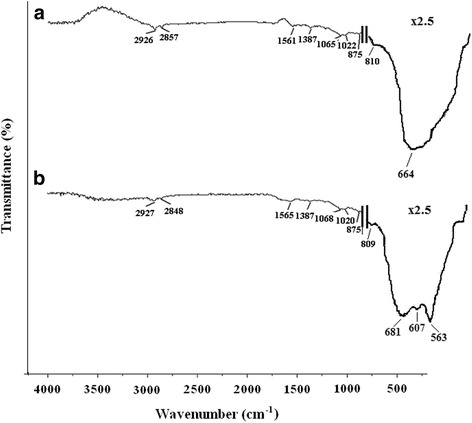
IR spectra of tin (IV) oxide samples. **a** SnO_2_ synthesized at faster heating rate of 80 K/min (TO1). **b** SnO_2_ synthesized at slower heating rate of 20 K/min (TO2)

The infrared spectra of two SnO_2_ nanostructured samples are shown in Fig. 6. It is known from the analysis of literature (Table 2) that the vibrational bands of the SnO_2_ are divided into different regions where both vibrations of SnO_2_ atomic structure and vibration of absorbed species such as O_2_, CO_2_, and even H_2_O could be detected. The stretching and antisymmetric Sn–O, Sn–O–Sn, and O–Sn–O vibrations of SnO_2_ can be found in the range of 400–1050 cm^−1^, while absorbed O_2_ and CO_2_ molecule vibrations are located between 1050 and 3000 cm^−1^ region and physically absorbed water vibrational bands could be found at 3390–3413 cm^−1^ (Table 2). IR spectra presented in Fig. 6 correspond well with the results published in the literature, especially since the 563 cm^−1^ band present in the 1D SnO_2_ nanostructure in the current study was also reported to exist in 1D structures by others [23]. The presence of this extra absorption band in the 560–570-cm^−1^ region is known as a characteristic feature of 1D SnO_2_ structures, but the nature of their presence still requires clarifications.

**Table 2:** Absorption spectra of synthesized SnO_2_ samples

Wavenumber (cm^−1^)		Vibrational band	Reference data (cm^−1^)
TO1	TO2		
435	434	Sn–O (O–Sn–O)	428 [23]
–	563	Sn–O (Sn–OH)	537 [28], 546 [29], 567 [23]
–	607	Sn–O (Sn–O–Sn)	613 [30], 623 [31]
664	684	Sn–O	673 [23]
810	809	O–Sn–O	817 [32]
875	875	O–Sn–OH	866 [33]
1022	1020	Sn–O (O–Sn–O)	1021 [34]
1065	1068	O_2_^−^ (chemical adsorption)	1045 [34]
1387	1387	CO_2_ (physical adsorption)	1386 [35]
1561	1565	O_2_ (physical adsorption)	1580 [34]
–	–	H_2_O (bounded) (Sn–OH)	1631 [31], 1633 [31]
2857	2848	CO_2_ (chemical adsorption)	2840, 2925 [34]
2926	2927		
–	–	H_2_O (adsorbed) (Sn–OH)	3394 [28, 31], 3413 [28]

### I–U Measurements

**Fig. 7:**
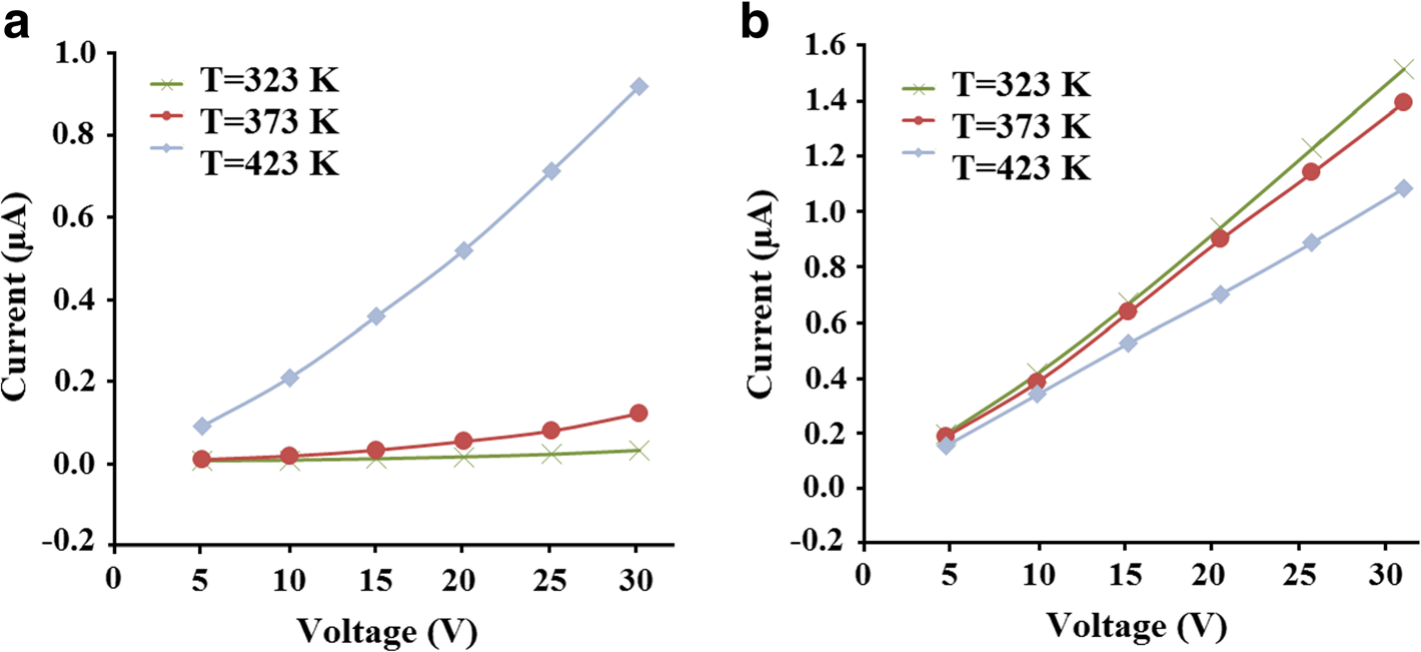
I–V curves of SnO_2_ samples. **a** SnO_2_ synthesized at faster heating rate of 80 K/min (TO1). **b** SnO_2_ synthesized at slower heating rate of 20 K/min (TO2)

To study the influence of morphology on electrical properties, I–V curves for 0D and 1D SnO_2_ nanostructures were measured. Figure 7 shows I–V curves at 323, 373, and 423 K in range of 5–30 V.

As seen on Fig. 7, the current-voltage curves of these samples are different. For 0D SnO_2_ sample, I–V curves are non-ohmic at all temperatures while 1D tin (IV) oxide sample is characterized by linear (ohmic) current-voltage dependences. The various nature of curves for 0D and 1D nanostructures related to the different surface to volume ratios. Change in this ratio leads to a change in the I–V behavior of the material. It is known that both surface and bulk conductivities of the SnO_2_ contribute to the overall conductivity.

In addition, it is known that the ohmic behavior of current-voltage characteristics is very important for the sensing properties of the material, as the sensing properties of SnO_2_ are significantly improved if the material is showing ohmic type semiconducting behavior [27]. Therefore, 1D nanostructures are more desirable for use in gas sensors.

## Conclusions

The single crystalline particles of SnO_2_ of different morphology (zero-dimensional (0D) and one-dimensional (1D) nanostructures) were obtained by thermal evaporation method. Such significant difference in the morphology of the SnO_2_ nanostructures were achieved due to their different synthesis conditions, as it was found that slower heating rate during the thermal evaporation brings changes to the SnO_2_ morphology allowing to receive 1D nanofibers. The comparison of different properties of 0D and 1D SnO_2_ nanostructures is presented. It was determined that the morphology has significant impact on the structural and functional properties of SnO_2_ as it is reflected in changes in crystal structure where texture formation was recorded, variation of IR spectra, as well as different I–V characteristics of gas sensors based on 0D and 1D SnO_2_ structures. It was also established that considerable changes in behavior of SnO_2_ depends also on surface to volume ratios of nanostructures.

Based on the experimental data, 1D nanostructures are more desirable for use in gas sensors. Further comparative research of 0D and 1D nanostructures will be carried out regarding sensory properties.
